# Discrete Structure of the Brain Rhythms

**DOI:** 10.1038/s41598-018-37196-0

**Published:** 2019-01-28

**Authors:** L. Perotti, J. DeVito, D. Bessis, Y. Dabaghian

**Affiliations:** 1 Department of Physics, Texas Southern University, 3100 Cleburne Ave., Houston, Texas 77004 USA; 2 Department of Neurology, The University of Texas Health Science Center at Houston, Houston, TX 77030 USA

## Abstract

Neuronal activity in the brain generates synchronous oscillations of the Local Field Potential (LFP). The traditional analyses of the LFPs are based on decomposing the signal into simpler components, such as sinusoidal harmonics. However, a common drawback of such methods is that the decomposition primitives are usually presumed from the onset, which may bias our understanding of the signal’s structure. Here, we introduce an alternative approach that allows an impartial, high resolution, hands-off decomposition of the brain waves into a small number of discrete, frequency-modulated oscillatory processes, which we call oscillons. In particular, we demonstrate that mouse hippocampal LFP contain a single oscillon that occupies the *θ*-frequency band and a couple of *γ*-oscillons that correspond, respectively, to slow and fast *γ*-waves. Since the oscillons were identified empirically, they may represent the actual, physical structure of synchronous oscillations in neuronal ensembles, whereas Fourier-defined “brain waves” are nothing but poorly resolved oscillons.

## Introduction

Neurons in the brain are submerged into a rhythmically oscillating electrical field, created by synchronized synaptic currents^1^. The corresponding potential, known as local field potential (LFP) is one of the principal determinants of neural activity at all levels, from the synchronized spiking of the individual neurons to high-level cognitive processes^2^. Attempts to understand the structure and function of LFP oscillations, and of their spatiotemporally smoothed counterparts—the electroencephalograms (EEG), continues almost a century and a systematic understanding of their roles begins to take shape.

The possibility to identify true physiological functions of the LFP depends fundamentally on the mathematical and computational tools used for its analysis. The majority of the currently existing methods are based on breaking the signal into a combination of simpler components, such as sinusoidal harmonics or wavelets^3,4^, and then correlating them with physiological, behavioral and cognitive phenomena^5,6^. For example, wavelet analysis is most appropriate for studying time-localized events, such as ripples or spindles^7,8^, whereas for the general analyses, the oscillatory nature of LFPs suggests using discrete Fourier decomposition into a set of plane waves with a fixed set of frequencies *ω*, 2*ω*, 3*ω*, …. The latter approach has dominated the field for the last several decades and now constitutes, in effect, the only systematic framework for our understanding of the structure and the physiological functions of the brain rhythms^6^. However, a common flaw of these methods is that the decomposition primitives are presumed from the onset, and the goal of subsequent analyses reduces merely to identifying the combination that best reproduces the original signal. Since no method can guarantee a universally good representation of the signals’ features and since the physiological structure of the LFPs remains unknown, obtaining a physically adequate description of the brain rhythms is a matter of fundamental importance.

Below we propose a novel approach of LFP analysis based on a recent series of publications^9–11^, in which an optimal set of frequencies *ω*_1_,*ω*_2_, …, is estimated, at every moment of time *t*, using the Padé Approximation Theory^12^. In contrast with the Fourier method, these adaptively optimized values can freely change within the sampling frequency domain, guided only by the signal’s structure. The resulting harmonics are highly responsive to the signals’ dynamics and capture subtle details of the signal’s spectrum very effectively, as one would expect from a Padé Approximation based technique. We call the new method Discrete Padé Transform (DPT), to emphasize certain key correspondences with the traditional Discrete Fourier Transform (DFT).

Applying DPT analyses to LFP rhythms recorded in mouse hippocampi reveals a new level in their structure–a small number of frequency-modulated oscillatory processes, which we call *oscillons*. Importantly, oscillons are observed in the physiologically important theta (*θ*)^13–15^ and gamma (*γ*)^16,17^ frequency domains, but are much sharper defined. For example, in the Fourier approach, the *θ*-rhythm is loosely defined as a combination of the plane waves with frequencies between 4 and 12 Hz^13–15^. In contrast, our method suggests that there exists a *single* frequency-modulated wave—the *θ*-oscillon—that occupies the entire *θ* frequency band and *constitutes* the *θ*-rhythm. Similarly, we observe oscillons in the low and high *γ*-frequency domains. The superposition of the oscillons reproduces the original LFP signal with high accuracy, which implies that these waves provide a remarkably sparse representation of the LFP oscillations. Since oscillons emerged as a result of empirical analyses, we hypothesize that they represent the actual, physical structure of synchronized neuronal oscillations, which were previously approximately described as the Fourier-defined “brain waves.”

## Results

### The oscillons

We implemented a “Short Time Padé Transform” (STPT), in which a short segment of the time series (that fits into a window of a width *T*_*W*_) is analyzed at a time. This allows us to follow the signal’s spectral composition on moment-to-moment basis and to illustrate its spectral dynamics using Padé spectrograms (the analogues of to the standard Fourier spectrograms^18,19^).

**Figure 1:**
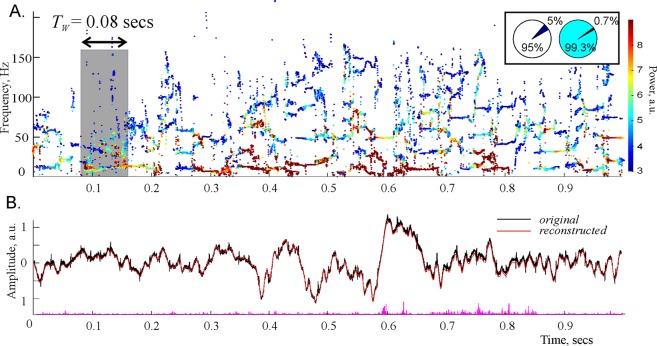
Padé spectrograms of the hippocampal LFP signal. (**A**) Discrete Padé Spectrogram (DPS) produced for the LFP signal recorded in the CA1 region of the rodent hippocampus at the sampling rate 10 kHz. At each moment of time, the vertical cross section of the spectrogram gives the instantaneous set of the regular frequencies. At consecutive moments of time, these frequencies produce distinct, contiguous traces, which can be regarded as timelines of discrete oscillatory processes—the spectral waves with varying frequencies *ω*_*q*_(*t*), amplitudes *A*_*q*_(*t*) (shown by the color of dots) and phases *ψ*_*q*_(*t*) (not shown). Note that the higher frequency spectral waves tend to have lower amplitudes. Highest amplitudes appear in the *θ*-region, i.e. in the frequency range between 4 and 12 Hz. The spectral waves above 100 Hz tend to be scarce and discontinuous, representing time-localized splashes of LFP. The width of the time window is *T*_*W*_ = 0.08 sec (800 data points). The pie diagrams in the box show that stable harmonics constitute only 5% of their total number, but carry over 99% of the signal’s power. (**B**) The LFP signal reconstructed from the regular poles (red trace) closely matches the original signal (black trace) over its entire length, which demonstrates that the oscillon decomposition (2) provides an accurate representation of the signal. The difference between the original and the reconstructed signal is due to the removed noise component—the discarded “irregular” harmonics (the magenta “grass” along the *x*-axis). Although their number is large (about 90–99% of the total number of frequencies), their combined contribution is small—only about 10^−3^–10^−4^% of the signals power.

Applying these analyses to the hippocampal LFPs recorded in awake rodents during habituation stage^20^, we observed that there exist two types of time-modulated frequencies (Fig. 1). First, there is a set of frequencies that change across time in a regular manner, leaving distinct, continuous traces—the *spectral waves*. As shown on Fig. 1A, the most robust, continuous spectral waves with high amplitudes (typically three or four of them) are confined to the low frequency domain and roughly correspond to the traditional *θ*- and *γ*-waves^13,16^. The higher frequency (over 100 Hz) spectral waves are scarce and short, representing time-localized oscillatory phenomena that correspond, in the standard Fourier approach, to fast *γ* events^21^, sharp wave ripples (SWRs)^22^ or spindles^23^. Second, there exists a large set of “irregular” frequencies that assume sporadic values from one moment to another, without producing contiguous patterns and that correspond to instantaneous waves with very low amplitudes.

From the mathematical perspective, the existence of these two types of instantaneous frequencies can be explained based on several subtle theorems of Complex Analysis, which point out that the “irregular” harmonics represent the signal’s noise component, whereas the “regular,” stable harmonics define its oscillatory part (see^24–27^ and the Mathematical Supplement). Thus, in addition to revealing subtle dynamics the frequency spectrum, the DPT method allows a context-free, impartial identification of noise, which makes it particularly important for the biological applications^28,29^.

As it turns out, the unstable, or “noisy,” frequencies typically constitute over 95% of the total number of harmonics (Fig. 1A). However, the superposition of the harmonics that correspond to the remaining, *stable* frequencies captures the shape of the signal remarkably well (Fig. 1B). In other words, although only a small portion of frequencies are regular, they contribute over 99% of the signal’s amplitude: typically, the original LFP signal differs from the superposition of the stable harmonics by less than 1%. If the contribution of the “irregular” harmonics (i.e., the noise component *ξ*(*t*)) is included, the difference is less than 10^−4^–10^−6^ of the signal’s amplitude.

These results suggest that the familiar Fourier decomposition of the LFP signals into a superposition of plane waves with *constant* frequencies,

1 $$r(t)={{\rm{\Sigma }}}_{p=1}^{N}\,{a}_{p}{e}^{i{\omega }_{p}t},$$

should be replaced by a combination of a few phase-modulated waves embedded into a weak noise background *ξ*(*t*),

2 $$s(t)={{\rm{\Sigma }}}_{q=1}^{M}\,{A}_{q}{e}^{i{\varphi }_{q}(t)}+\xi (t),$$

which we call *oscillons*. We emphasize that the number $M\ll N$ of the oscillons in the decomposition (2), their amplitudes *A*_*q*_, their phases *ϕ*_*q*_ and the time-dependent frequencies *ω*_*q*_(*t*) = ∂_*t*_*ϕ*_*q*_(*t*) (i.e., the spectral waves shown on Fig. 1A) are reconstructed on moment-by-moment basis from the local segments of the LFP signal in a hands-off manner: we do not presume *a priori* how many frequencies will be qualified as “stable,” when these stable frequencies will appear or disappear, or how their values will evolve in time, or what the corresponding amplitudes will be. Thus, the structure of the decomposition (2) is obtained *empirically*, which suggests that the oscillons may reflect the actual, physical structure of the LFP rhythms.

### The spectral waves

**Figure 2:**
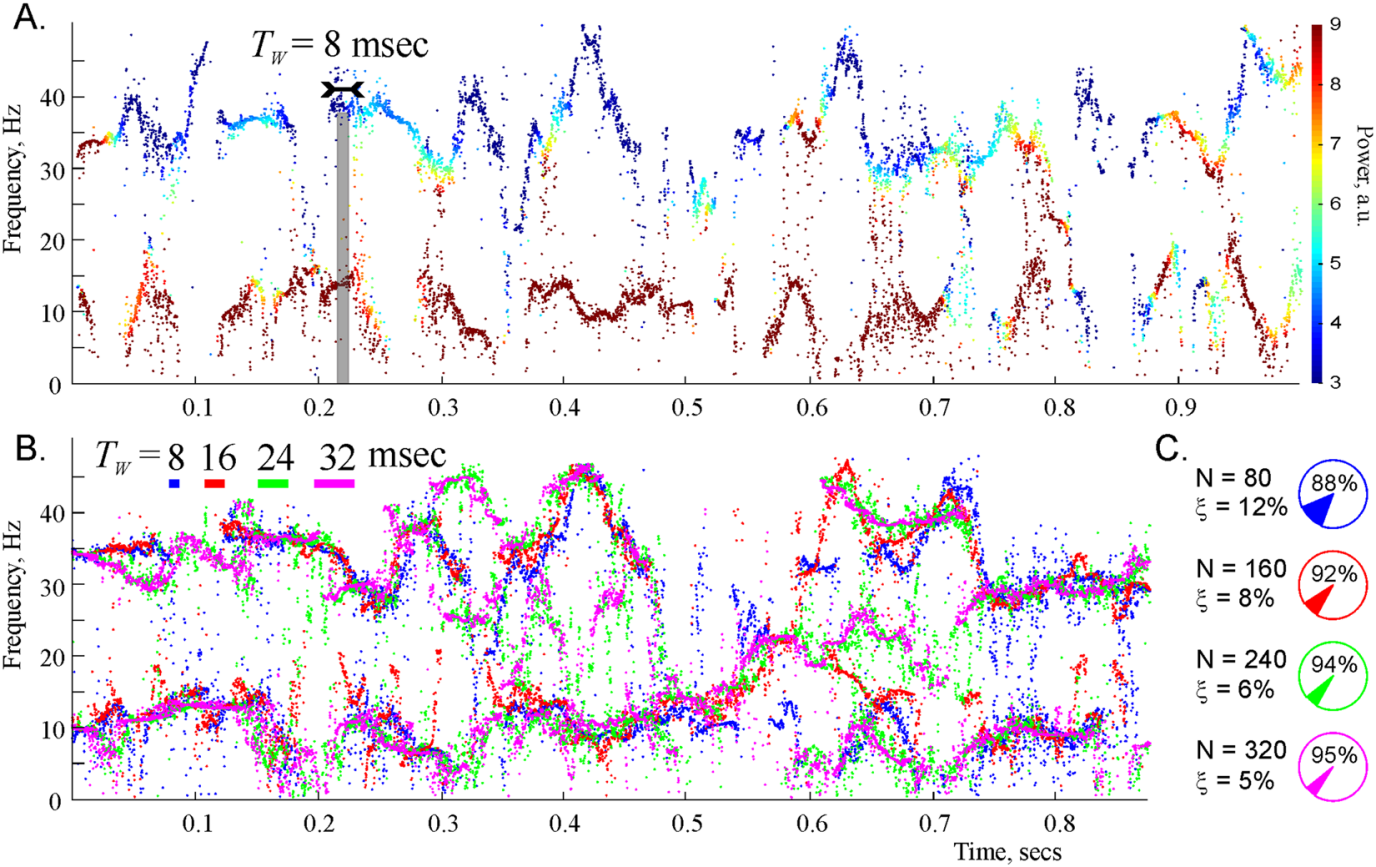
Spectral waves. (**A**) A detailed representation of the lower portion the spectrogram recomputed for *T*_*W*_ = 0.08 sec (80 data points) exhibits clear oscillatory patterns. (**B**) The shape of the two lowest frequency spectral waves is stable with respect to the variation of time window size, *T*_*W*_. The strikes of different color in the top left corner represent the widths of the four *T*_*W*_-values used in DPT analysis. The corresponding reconstructed frequencies are shown by the dots of the same color. Although the frequencies obtained for different *T*_*W*_s do not match each other exactly, they outline approximately the same shape, which, we hypothesize, reflects the physical pattern of synchronized neuronal activity that produced the analyzed LFP signal. (**C**) Pie diagrams illustrate the numbers of data points *N* = 80, *N* = 160, *N* = 240, *N* = 320 and the mean numbers of the regular and the irregular (noisy) harmonics in each case.

We studied the structure the two lowest spectral waves using high temporal resolution spectrograms (Fig. 2A). Notice that these spectral waves have a clear oscillatory structure,

3 $${\omega }_{q}(t)={\omega }_{q,0}+{\omega }_{q,1}\,\sin ({{\rm{\Omega }}}_{q,1}t+{\phi }_{q,1})+{\omega }_{q,2}\,\sin ({{\rm{\Omega }}}_{q,2}t+{\phi }_{q,2})+\ldots ,\,q=1,2,$$

characterized by a mean frequency *ω*_*q*,0_, as well as by the amplitudes, *ω*_*q*,*i*_, the frequencies, Ω_*θ*,*i*_, and the phases, *φ*_*θ*,*i*_, of the modulating harmonics. The lowest wave has the mean frequency of about 8 Hz and lies in the domain 2 ≤ *ω*/2*π* ≤ 17 Hz, which corresponds to the *θ*-frequency range^13^. The second wave has the mean frequency of about 35 Hz and lies in the low-*γ* domain 25 ≤ *ω*/2*π* ≤ 45 Hz^16^. Importantly, the spectral waves are well separated from one another: the difference between their mean frequencies is larger than their amplitudes, which allows indexing them using the standard brain wave notations, as *ω*_*θ*_(*t*) and ${\omega }_{{\gamma }_{l}}(t)$ respectively, e.g.,

4 $${\omega }_{\theta }(t)={\omega }_{\theta ,0}+{\omega }_{\theta ,1}\,\sin ({{\rm{\Omega }}}_{\theta ,1}t+{\phi }_{\theta ,1})+{\omega }_{\theta ,2}\,\sin ({{\rm{\Omega }}}_{\theta ,2}t+{\phi }_{\theta ,2})+\ldots ,$$

for the *θ* spectral wave an

5 $${\omega }_{{\gamma }_{l}}(t)={\omega }_{{\gamma }_{l},0}+{\omega }_{{\gamma }_{l},1}\,\sin ({{\rm{\Omega }}}_{{\gamma }_{l},1}t+{\phi }_{{\gamma }_{l},1})+{\omega }_{{\gamma }_{l},2}\,\sin ({{\rm{\Omega }}}_{{\gamma }_{l},2}t+{\phi }_{{\gamma }_{l},2})+\ldots $$

for the low-*γ* spectral wave, etc.

We verified that these structures are stable with respect to the variations of the STPT parameters, e.g., to changing the sliding window size, *T*_*W*_. The size of the sliding window, and hence the number of points *N* that fall within this window can be changed by over 400%, without affecting the overall shape of the spectral waves (Fig. 2B). The smallest window size (a few milliseconds) is restricted by the requirement that the number of data points captured within *T*_*W*_ should be bigger than the physical number of the spectral waves. On the other hand, the maximal value of *T*_*W*_ is limited by the temporal resolution of STPT: if the size of the window becomes comparable to the characteristic period of a physical spectral wave, then the reconstructed wave looses its undulating shape and may instead produce a set of sidebands surrounding the mean frequency^3^. This effect limits the magnitude of the *T*_*W*_ to abut 50 milliseconds—for larger values of *T*_*W*_, the undulating structure begins to straighten out, as shown on Fig. 1A for *T*_*W*_ = 80 msec.

In contrast with this behavior, the values of the irregular frequencies are highly sensitive to the sliding window size and other DPT parameters, as one would expect from a noise-representing component. The corresponding “noisy” harmonics can therefore be easily detected and removed using simple numerical procedures (see Mathematical Supplement). Moreover, we verified that the structure of the Padé Spectrogram, i.e., the parameters the oscillons remain stable even if the amount of numerically injected noise exceeds the signal’s natural noise level by an order of magnitude (about 10^−4^ of the signal’s mean amplitude), which indicates that the oscillatory part of the signal is robustly identified.

### Parameters of the low frequency oscillons

**Figure 3:**
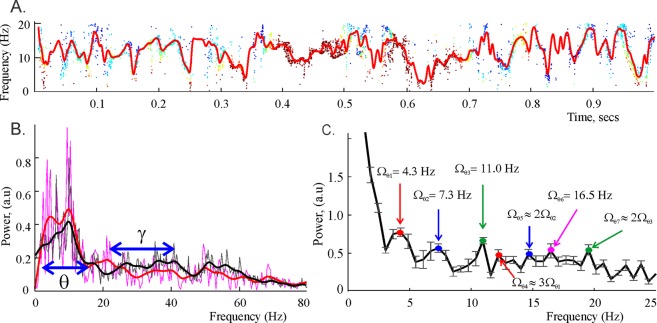
Parameters of the spectral waves. (**A**) The red curve shows the smoothened *θ* spectral wave, obtained by interpolating the “raw” trace of the reconstructed frequencies shown on Fig. 2A over the uniformly spaced time points. (**B**) The power spectra produced by the Discrete Padé decomposition (DPT, red) and the standard Discrete Fourier decomposition (DFT, black) exhibit characteristic peaks around the mean frequency of the *θ*-oscillon, *ω*_*θ*,0_/2*π* ≈ 7.5 Hz. The height of the peaks defines the amplitudes, respectively, of the *θ*-oscillon in the DPT approach and of the *θ*-rhythm in DFT. A smaller peak at about 34 Hz corresponds to the mean frequency of the low *γ* oscillon, ${\omega }_{{\gamma }_{l\mathrm{,0}}}/2\pi \approx 34$. The *θ* and the low *γ* frequency domains, marked by blue arrows, are defined by the amplitudes of the corresponding spectral waves. (**C**) The smoothened waves are used to compute the DFT transform and to extract the modulating frequencies Ω_*θ*,1_ ≈ 4.3 Hz, Ω_*θ*,2_ ≈ 7.3 Hz, Ω_*θ*,3_ ≈ 11 Hz, …, of the decomposition (4–5). The error margin in most estimates is ±0.5 Hz. Notice that there exist several approximate resonant relationships, e.g., Ω_*θ*,4_ ≈ 3Ω_*θ*,1_, Ω_*θ*,5_ ≈ 2Ω_*θ*,2_ and Ω_*θ*,7_ ≈ Ω_*θ*,3_, which suggest that the spectral *θ*-wave contains higher harmonics of a smaller set of prime frequencies.

To obtain a more stable description of the underlying patterns, we interpolated the spectral waves over the uniformly spaced time points (Fig. 3A) and then studied the resulting “smoothened” spectral waves using the standard DFT tools. In particular, we found that, for studied LFP signals, the mean frequency of the *θ*-oscillon is about *ω*_*θ*,0_/2*π* = 7.5 ± 0.5 Hz and the mean frequency of the low *γ*-oscillon is ${\omega }_{{\gamma }_{l,0}}/2\pi =34\pm 2$ Hz, which correspond to the traditional (Fourier defined) average frequencies of the *θ* and the low *γ* rhythms.

The amplitudes of the *θ* and the low *γ* spectral waves—7.0 ± 1.5 Hz and 10.1 ± 1.7 Hz respectively—define the frequency domains (spectral widths) of the *θ* and the low *γ* rhythms (Fig. 3B). The amplitudes of the corresponding oscillons constitute approximately *A*_*θ*_/*A* ≈ 62% and ${A}_{{\gamma }_{l}}/A\approx \mathrm{17 \% }$ of the net signals’ amplitude *A*, i.e., the *θ* and the low *γ* oscillons carry about 80% of the signals’ magnitude.

The oscillatory parts of the spectral waves are also characterized by a stable set of frequencies and amplitudes: for the first two modulating harmonics we found *ω*_*θ*,1_/2*π* ≈ 4.3 Hz, *ω*_*θ*,2_/2*π* ≈ 3.2 Hz for the *θ* spectral wave (4) and ${\omega }_{{\gamma }_{l\mathrm{,1}}}/2\pi \approx 6.1$ Hz, ${\omega }_{{\gamma }_{l\mathrm{,2}}}/2\pi \approx 4.3$ Hz for the *γ* spectral wave (5). The corresponding modulating frequencies for the *θ*-oscillon are Ω_*θ*,1_ = 4.3 ± 0.45 Hz, Ω_*θ*,2_ = 7.3 ± 0.48 Hz, …, (Fig. 3C). The lowest modulating frequencies for the *γ*-oscillon are slightly higher: ${{\rm{\Omega }}}_{{\gamma }_{l},1}=5.3\pm 0.41$ Hz, ${{\rm{\Omega }}}_{{\gamma }_{l},2}=8.3\pm 0.51$ Hz, …. In general, the modulating frequencies tend to increase with the mean frequency.

Importantly, the reconstructed frequencies sometimes exhibit approximate resonance relationships (Fig. 3C), implying that some of the higher order frequencies may be overtones of a smaller set of prime frequencies that define the dynamics of neuronal synchronization^30–32^.

## Discussion

**Figure 4:**
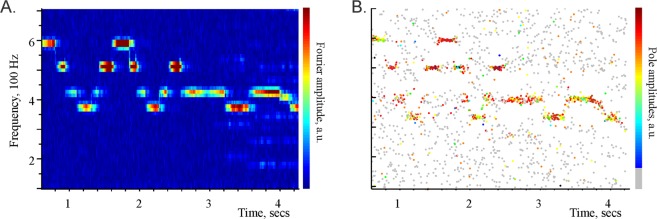
Correspondence between the Discrete Fourier (left) and Padé (right) spectral decompositions. (**A**) Fourier spectrogram of a 10 second long excerpt from C. Debussy’s Preludes, Book 1: No. 8. *La fille aux cheveux de lin*, in which the individual notes are clearly audible. The high amplitude streaks (colorbar on the right) correspond to the notes (D#5, B4, G4, F4, G4, B4, D5, B4, G4, F4, G4, B4, G4, F4, G4, F4, …). (**B**) The Discrete Padé spectrogram of the same signal. The frequencies produced by large amplitude poles (see colorbar on the right) match the frequencies of their Fourier counterparts shown on the left. The frequencies produced the Froissart doubles form a very low amplitude background “dust,” shown in gray. Our main hypothesis is that the oscillons detected in the LFP signals by the DPT method may be viewed as “notes” within the neuronal oscillations.

The Fourier and the Padé decompositions agree in simple cases, e.g., both spectrograms resolve the individual piano notes in a 10 sec excerpt from one of Claude Debussy’s Preludes (Fig. 4).

However, in more complex cases the DTP approach produces a more accurate description of the signal’s structure. For example, the Padé decomposition was previously used to detect faint gravitational waves in resonant interferometers, which were completely missed by the Fourier analyses^33^. In the case of the LFP signals, this method identifies a small number of structurally stable, frequency-modulated oscillons which may reflect the physical synchronization patterns in the hippocampal network.

Why these structures were not previously observed via Fourier method? The reason lies in the insufficient resolution of the latter, which is due to the well-known inherent conflict between the frequency and the temporal resolutions in Fourier Analysis^34^. Indeed, in order to observe changes in the signal’ spectrum, the size of the sliding time window, *T*_*W*_, should be *smaller* than the characteristic timescale of frequency’s change, *T*_*W*_ < Δ*T*. On the other hand, reducing *T*_*W*_ implies lowering the number of data points in the sliding window, which results in an equal reduction of the number of the discrete harmonics, in both the DFT and the DPT approaches. However, since in DFT method these harmonics are restricted to a rigid, uniformly distributed set of values (SFig. 1), a decrease in the number of data points necessarily results in an increase of the interval between neighboring discrete frequencies, i.e., in an unavoidable reduction of frequency resolution. In contrast, the DPT harmonics can move freely in the available frequency domain, responding to the spectral structure of the signal and providing a high resolution of the signals’ spectrum^11^. In other words, an increase in temporal resolution in DPT does not necessarily compromise the frequency resolution and vice versa, which allows describing the signal dynamics much more capably.

In the specific case illustrated on Fig. 2, the characteristic amplitudes of the spectral waves is about 15–25 Hz. Producing such frequency resolution in DFT at the sampling rate *S* = 10 kHz would require some *N* = 300–500 constant frequency harmonics, i.e., *N* = 300–500 data points, which can be collected over *T*_*W*_ = 30–50 msec time window. However, the characteristic period of the spectral waves is about 60 msec, which implies that for such *T*_*W*_s, the DFT will not be able to resolve the frequency wave dynamics and will replace it by an average frequency with some sidebands (see Mathematical Supplement). In contrast, a DPT that uses as few as 80 data points in a *T*_*W*_ = 8 msec wide time window, reliably capturing the shape of the spectral wave, which then remains overall unchanged as *T*_*W*_ increases fourfold.

Another key property of the DPT method is the intrinsic marker of noise, which is particularly important in biological applications^28,29^. In general, the task of distinguishing “genuine noise” from a “regular, but highly complex” signal poses not only a computational, but also a profound conceptual challenge^35,36^. In contrast with the standard *ad hoc* approaches, the DPT method allows a context-free, impartial identification of the noise component, as the part of the signal represented by the irregular harmonics.

The new structure also dovetails with the theoretical views on the origins of the LFP oscillations as on a result of synchronization of the neuronal spiking activity in both the excitatory and inhibitory networks^30–32^. Broadly speaking, it is believed that the LFP rhythms are due to a coupling between the electromagnetic fields produced by local neuronal groups^1^. If the coupling between these groups is sufficiently high, then the individual fields oscillating with amplitudes *a*_*p*_ and phases *x*_*p*_ synchronize, yielding a nonzero mean field ${{\rm{\Sigma }}}_{p}\,{a}_{p}{e}^{i{x}_{p}}=A{e}^{i\varphi }$ that is macroscopically observed as LFP^30–32^. In particular, the celebrated Kuramoto Model^30^ describes the synchronization between oscillators via a system of equations

6 $${\partial }_{t}{x}_{q}={\omega }_{q,0}+K\,{{\rm{\Sigma }}}_{p}\,\sin ({x}_{q}-{x}_{p}),$$

according to which the oscillators transit to a synchronized state, as the coupling strength *K* increases. Eq. (6) directly points out that the synchronized frequency, *ω*(*t*) = ∂_*t*_*ϕ*, should have the form (3). However, this form of expansion has not been previously extracted from the experimental data, which may be due to the fact that the Fourier method does not resolve the spectral structure in sufficient detail (SFig. 2). In contrast, the description of the LFP oscillations produced by the DPT method may provide such resolution and help to link the empirical data to theoretical models of neuronal synchronization.

## Supplementary information

Mathematical Supplement
