# Supplementary material for: Discrete Structure of the Brain Rhythms
Source: Sci Rep. 2019 Jan 28;9:1105. doi: 10.1038/s41598-018-37196-0 (PMC6349927; doi:10.1038/s41598-018-37196-0)
Supplement: Supplementary file 1 — Mathematical Supplement [file 41598_2018_37196_MOESM1_ESM.pdf]

Discrete Structure of the Brain Rhythms: Mathematical Supplement

L. Perotti[1], J. DeVito[2], D. Bessis[1], Y. Dabaghian[2*]

The Discrete Fourier Transform (DFT) is used to represent a given time series as a superposition of discrete harmonics with *fixed* frequencies (SFig. 1A). To that end, N recorded values, $s_1, s_2, \ldots, s_N$, are convolved with a set of N discrete harmonics, $z_l = e^{i2\pi l/N}$,

$$A_l = \Sigma_n s_n z_l^n. \tag{1}$$

The magnitude of this convolution defines the amplitude of the discrete plane wave z_l in the discrete Fourier decomposition: the most prominent oscillatory components produce peaks in the Fourier transform, whereas the noise broadens these peaks, lowers their magnitudes and generally obscures the spectral properties of the signal [1]. Similar effects are produced by the signal's nonstationarity, i.e., by the time dependence of the signal's frequencies.

The Discrete Padé Transform (DPT) method discussed here is based on studying the generating function of the recorded time series,

$$G(z) = \Sigma_n s_n z^n, \tag{2}$$

where $z = x + iy$ is a complex variable (i.e., the series expansion (2) is an extension of (1) into the entire complex plane), and of its Padé approximant—a ratio of two polynomials $P_{N-1}(z)$ and $Q_N(z)$,

$$G_N(z) = P_{N-1}(z)/Q_N(z) \tag{3}$$

that approximates $G(z)$ to the $2N$-th order of z [2].

Oscillatory component. In the analyses of the oscillatory signals, the N roots z_p, $p = 1, ..., N$, of the polynomial $Q_N(z)$—the poles of the Padé approximant—play the role of the discrete Fourier harmonics, z_l, in the DFT: they capture the spectral structure of the signal [3–5]. Indeed, consider a signal $r(t)$ obtained as a superposition of N_P damped oscillators,

$$r(t) = \Sigma_p A_p e^{-\alpha_p t}\cos(\omega_p t + \varphi_p), \tag{4}$$

where A_p is the amplitude of the pth oscillator with a damping exponent α_p, frequency ω_p and phase φ_p. If the signal is sampled at a frequency S, then it generates a discrete time series,

$$r_k = \Sigma_{p=1}^{N_P} c_p e^{i\omega_p^{(+)}k/S} + c_p^* e^{i\omega_p^{(-)}k/S}, \quad k \in \mathbb{Z}, \tag{5}$$

where $\omega^{(\pm)} = i\alpha_p \pm \omega_p$ and $c_p = A_p e^{i\varphi_p/2}$. The generating function of this series,

$$R(z) = \Sigma_{k=1}^{\infty} r_k z^k = \Sigma_{p=1}^{N_P}\left(\frac{c_p}{1 - ze^{i\omega_p^{(+)}/S}} + \frac{c_p^*}{1 - ze^{i\omega_p^{(-)}/S}}\right), \tag{6}$$

is a rational fraction of degree $(2N_P - 1)/2N_P$ with poles

$$z_p^{(\pm)} = e^{-i\omega_p^{(\pm)}/S}.$$

The phase of each pole z_p defines the frequency ω_p, and its magnitude defines the damping constant α_p. The residue of z_p defines the amplitude A_p and the phase φ_p of the corresponding oscillator.

Notice that poles come in complex conjugate pairs and have to lie either outside of the unit circle, if the signal is damped ($\Im\omega_p = \alpha_p > 0$), or on the circle if the signal has no damping.

Noise component. If a signal is perturbed by an additive noise $\xi(t)$, then the generating function (3) of the resulting "noisy" time series $s_n = r_n + \xi_n$ is the sum of the "regular" and the "noisy" part, $G(z) = R(z) + \Xi(z)$, where

$$\Xi(z) = \Sigma_n \xi_n z^n. \quad (7)$$

A remarkable theorem proven by H. Steinhaus [8] establishes that the poles of $\Xi(z)$ concentrate, with probability 1, at the unit circle (SFig. 1B). In other words, the generating function of a random data series is an analytic function inside the unit disk, possessing a dense set of poles as $|z|$ approaches 1. Thus, the total generating function of the full signal $G(z) = R(z) + \Xi(z)$ has a finite number of poles contributed by $R(z)$ and an infinite number of poles contributed by $\Xi(z)$.

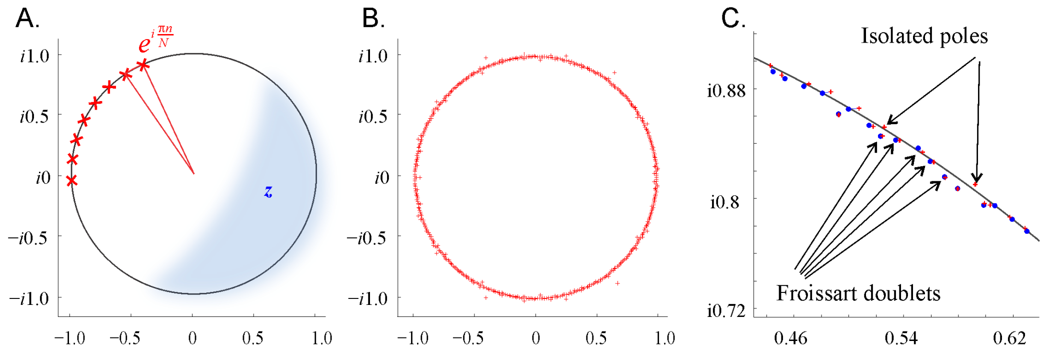

Figure 1: **Fourier and Padé in complex plane**. **A**. The discrete waves, $z_l = e^{i2\pi l/N}$, used to construct the Fourier decompositions, are uniformly distributed over the unit circle S^1, embedded into the complex plane of the variable z. **B**. The poles of the Padé approximant to the signal's generating function, z_p (red crosses), also concentrate in a close vicinity of the unit circle, in accordance with Steinhaus' theorem [8]. For illustration purposes, the number of Padé-poles shown on panel B is much larger than the number of harmonics shown on panel A. These poles are not constrained to any *a priori* selected locations; in fact, their positions in the complex plane C^1 are dictated solely by the signal's structure, which ultimately leads to the super-resolution property [5–7]. **C**. According to the Froissart's theory [3, 9], zeros and poles that represent the noise component of the signal form close pairs—the Froissart doublets. A zoom-in into a small segment of the unit circle shows many Froissart doublets (zeros shown as blue dots), and two isolated poles that represent the regular, oscillatory part of the signal.

A key property of the Padé approximant to $\Xi(z)$ is that its poles occur in close vicinities of its zeros, forming the so-called *Froissart doublets* [9–11] that can be easily detected numerically (Fig. 1C). In our analyses, the typical distance in a pole-zero pair is smaller than $10^{-6} - 10^{-7}$ in the standard Euclidean metric on C^1. We hence identified such pairs as the ones smaller than a critical distance $\delta = 10^{-5}$. These results are stable: injecting small amounts of white and colored noise into the signal (about 10^{-3} of the signal's mean amplitude, i.e., at least ten times more than the signal's natural noise level) does not alter the reconstructed positions of the regular poles and hence the parameters the spectral waves remain the same as the "perturbed" Froissart doublets are removed.

***J*-matrix formalism**. In order to obtain a Padé approximation to $G(z)$ in the entire complex plane, the $\Xi(z)$ has to be analytically extend through its natural boundary, which remains an open problem of complex analysis. However, the "J-matrix approach" developed in a recent series of publications [3–5], allows addressing this problem in practical terms. The generating function $G(z)$ can be associated with a tri-diagonal Hilbert space operator J that has $G(z)$ as its resolvent matrix

element, $G(z) = \langle e_0|(J - z1)^{-1}|e_0\rangle$, $e_0 = (1, 0, ...)$ [2]. In accordance with Steinhaus' theorem, the spectrum of J consists of two parts: an essential spectrum with support on the unit circle, which represents the noise component and a discrete spectrum, containing a finite number of poles outside the unit circle, which represent the regular component of the signal (a finite number of damped oscillators). In the spectrum of finite order truncations J_N of the J-operator, the poles of the Froissart doublets take the place of the essential spectrum. Moreover, these finite matrices can be explicitly constructed as follows. Let us consider the set of subdiagonal Padé approximations to the generating function of a given time series defined by (3). The polynomials $Q_N(z)$ satisfy a third order recursive relation which can be written in a matrix form, $J_N V = zV$ where J_N *is the (tri-diagonal) finite order matrix approximation to the J-operator of order* $N + 1$. The column vector V is defined by the polynomials Q_N, $V_T = [Q_0(z), Q_1(z), ..., Q_N(z)]$. The zeros of $Q_{N+1}(z)$ define the eigenvalues $(z_0, z_1, ..., z_N)$ of J_N and therefore the poles of R_N. The same procedure applied to P_N (with a slightly modified matrix) gives us the zeros of G_N, thus completely characterizing R_N itself.

Short Time Padé Transform, (STPT) is analogous to the standard Short Time Fourier Transform (STFT) method [12]. Starting with a segment $s_1, s_2, ..., s_N$ centered at t_1, we compute the Padé approximants, identify and discard the Froissart doublets, and then evaluate the frequencies, $\omega_q(t_1)$, the amplitudes, $A_q(t_1)$ and the phases, $\varphi_q(t_1)$, associated with the stable poles $z_1, z_2, ...z_{p_1}$, $q = 1, ..., p_1$. After that, the window is shifted by ΔT to the position centered at t_2, and the same analysis is applied to the next segment of the time series, revealing the frequencies, $\omega_i(t_2)$, the amplitudes, $A_i(t_2)$ and the phases, $\varphi_i(t_2)$, $i = 1, ..., p_2$, and so on.

Separating the noise from the oscillations. The Froissart doublets and the regular poles of $G(z)$, exhibit qualitatively different behaviors in response to changes of the DPT algorithms' parameters. If the size of time window T_W in the STPT is altered, or as it is shifted from one segment of the time series to another, or if the order of the Padé approximant is changed, the Froissart-paired poles move significantly and irregularly around the unit circle, as one would expect from a structure that represents noise. In contrast, the poles associated with the regular part of the signal remain stable and isolated. These differences can be easily detected numerically, producing the computational DPT method [3, 4].

Note that every data point obtained by the STPT method is obtained independently: evaluation of N frequencies at each time-step, identification of the "noisy" vs. "regular" frequencies, etc. does not affect the values obtained at the other time-steps and hence the pattern formed by the data points is completely empirical.

Computing the parameters of the spectral waves. Since the instantaneous parameters of the oscillons are computed independently based on a finite number of data points, the reconstructed spectral waves contain gaps and other irregularities. We therefore construct the smoothened spectral waves by interpolating the "raw" traces of the regular frequencies over the uniformly spaced time points, and compute the mean parameters $\omega_{q,0}$, $\omega_{q,i}$, $\Omega_{q,i}$, and $\varphi_{q,i}$ in the expansion

$$\omega_q(t) \equiv \partial_t \phi_q = \omega_{q,0} + \omega_{q,1} \sin(\Omega_{q,1} t + \varphi_{q,1}) + \omega_{q,2} \sin(\Omega_{q,2} t + \varphi_{q,2}) + \dots \quad (8)$$

using the standard DFT methods.

The SFig. 2 illustrates how the inherent conflict between the time and the frequency resolutions obscures the spectral waves in the Fourier spectrogram.

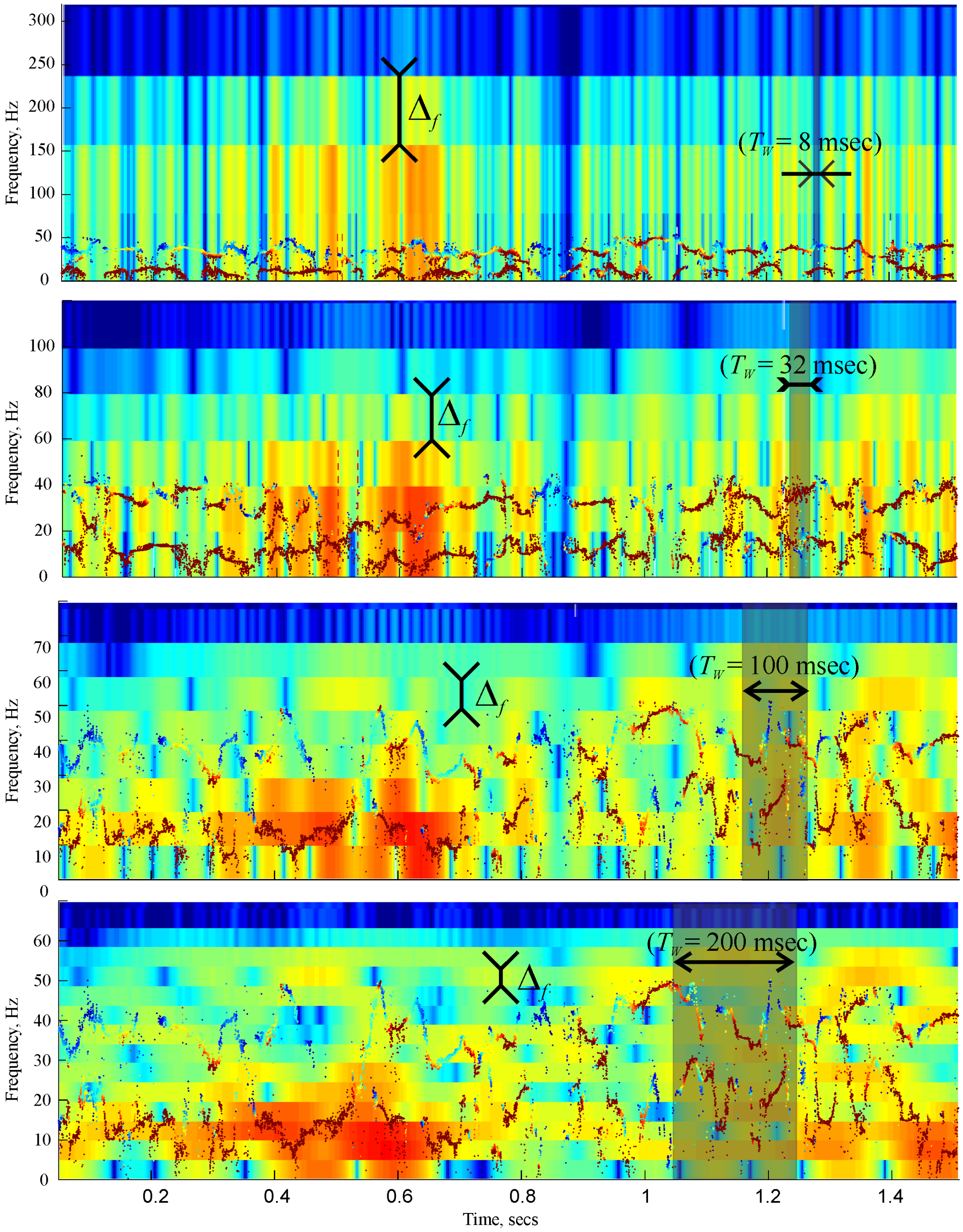

Figure 2: **Insufficiency of Fourier resolution**. The two lowest (θ and low-γ) spectral waves of an LFP signal, filtered between 1 and 56 Hz, are superimposed on four Fourier spectrograms of the same signal, computed for $T_W = 8$ msec, $T_W = 32$ msec, $T_W = 100$ msec and $T_W = 200$ msec. The sliding window widths T_W are shown by gray vertical stripes. The horizontal stripes on each Fourier spectrogram indicate the magnitude of the spectral resolution, Δ_f. The spectral resolution of the Fourier spectrogram becomes comparable to the frequency scale of the spectral waves only for $T_W = 100$ msec (third panel from the top), but the temporal resolution at this value exceeds the characteristic period of the spectral waves. Increasing the frequency resolution broadens the window size beyond the spectral waves' period (bottom panel) and vice versa, increasing temporal resolution destroys the frequency resolution (top two panels). As a result, the spectral waves remain unresolved by the DFT, which can only detect a band of increased amplitudes, but not the detailed pattern of the oscillating frequencies.

The wavelet spectrograms (scalograms) of the same signal using three different wavelets are shown on SFig. 3.

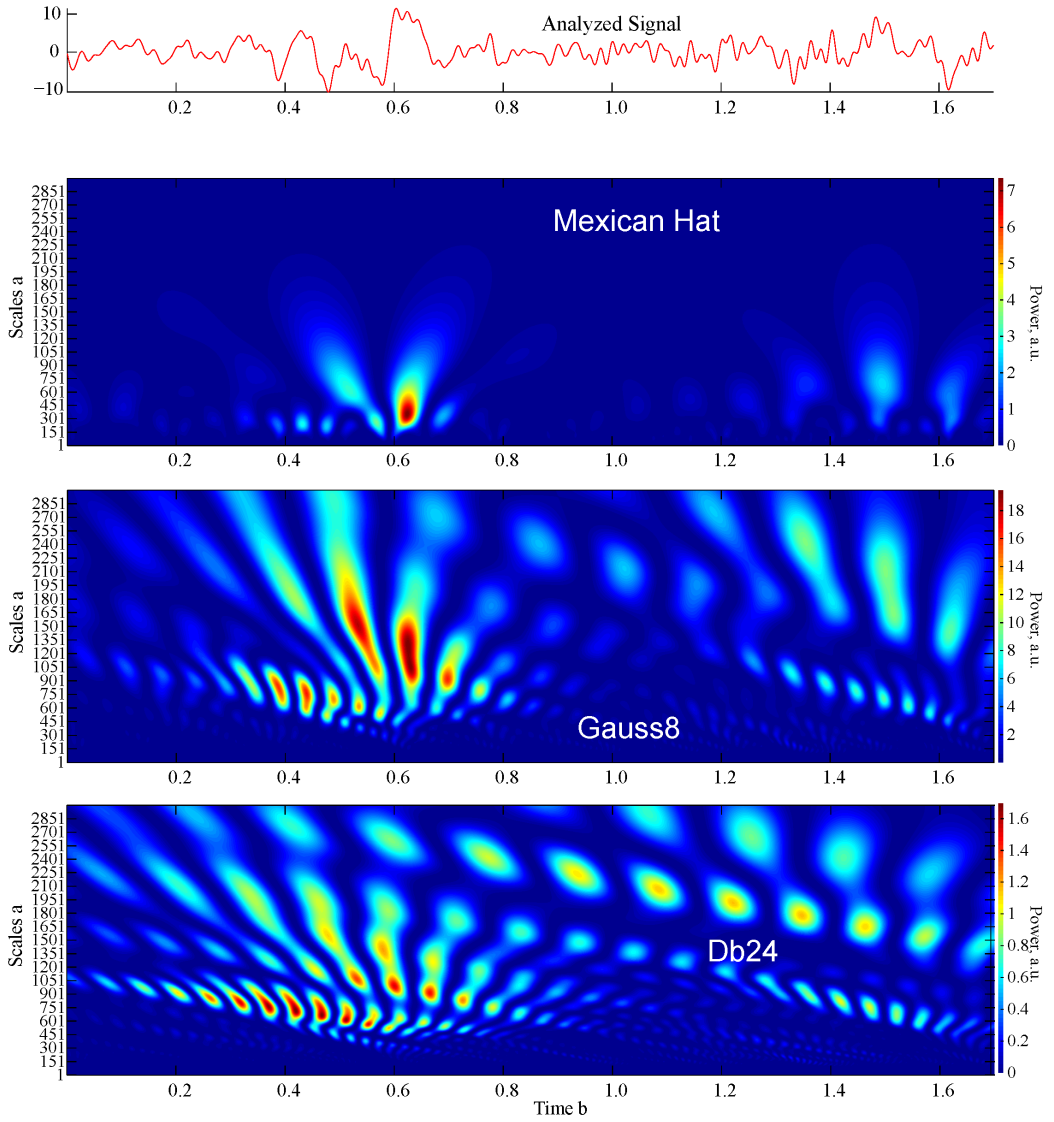

Figure 3: **Wavelet spectrograms** of the same signal, computed for the "Mexican hat" (top panel), the Gauss wavelet of level 8 (middle panel) and Daubechies' wavelet of level 24 (bottom panel). In all three cases,the maxima of the wavelet coefficients correspond to undulating patterns of the signals' amplitude at different temporal scales but do not resolve the spectral waves.

To illustrate the effectiveness of the proposed method, we simulated a superposition of five artificial oscillons with the amplitudes $A_1 = 0.5$, $A_2 = 0.3$, $A_3 = 0.15$, $A_4 = 0.1$ and $A_5 = 0.05$, the mean frequencies are $\omega_{1,0} = 5$ Hz, $\omega_{2,0} \approx 20$ Hz, $\omega_{3,0} \approx 30$ Hz, $\omega_{4,0} \approx 40$ Hz, and $\omega_{5,0} \approx 50$ Hz, and five modulating frequencies $\omega_{1,1} = 2$ Hz, $\omega_{2,1} = 2.5$ Hz, $\omega_{3,1} = 4$ Hz, $\omega_{4,1} = 5$ Hz and $\omega_{2,1} = 6$ Hz respectively. The amplitudes of the frequency modulations are approximately π Hz in all cases. The resulting "spectral waves" are shown as black sinusoids in the background of the four panels of SFig. 4. Each panel corresponds to a particular window width: $T_W = 0.10$ sec, $T_W = 0.15$ sec, $T_W = 0.2$ sec and $T_W = 0.25$ sec, at the sampling rate of $S = 1000$ Hz. In the first case, the DPT is therefore based on $N_1 = 100$ data points per window, i.e., 50 sample frequencies

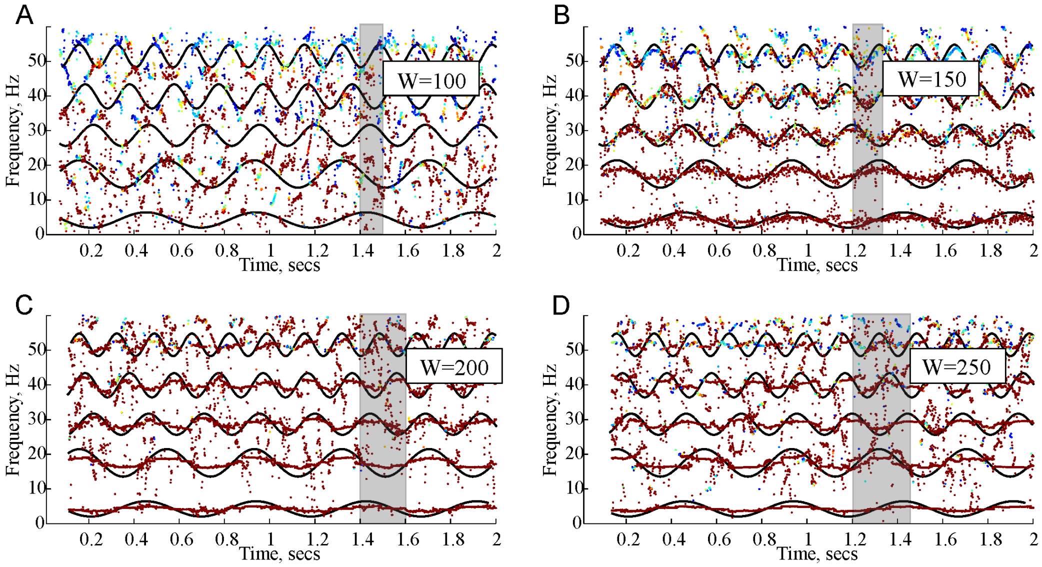

Figure 4: **Discrete Padé spectrograms** of the simulated combination of five oscillons, computed for four window widths ($T_W = 0.10$ sec, $T_W = 0.15$ sec, $T_W = 0.2$ sec and $T_W = 0.25$) . While the first window is too narrow to capture the structure of the spectral waves, the second window resolves them. The last two values of T_W are too large–the undulating pattern of the spectral waves is replaced by the emerging sidebands.

occupying the range between 0 and 500 Hz, or about one frequency per 10 Hz interval. As shown on the SFig. 4A, this (or smaller) values are insufficient for resolving oscillons with magnitude $\pm\pi$: the undulatory pattern is not captured. In the second case, each window contains $N_2 = 150$ points, or one frequency per $\approx$ 6.6 Hz, and the spectral waves become apparent (SFig. 4B). If window becomes bigger, $N_3 = 200$ points (SFig. 4C) or $N_4 = 250$ points (SFig. 4D), the temporal resolution suffers: the patterns of all spectral waves become averaged over the window width. As a result the upper spectral waves produce sidebands and the lowest spectral waves the flatten out. We emphasize however, that the original signal can be reconstructed with high precision in all cases; the issue is only whether the spectral can or cannot be resolved.

For comparison, the corresponding Fourier spectrograms and the wavelet scalogram computed using Daubechies' wavelet of level 24 are shown on SFig. 5.

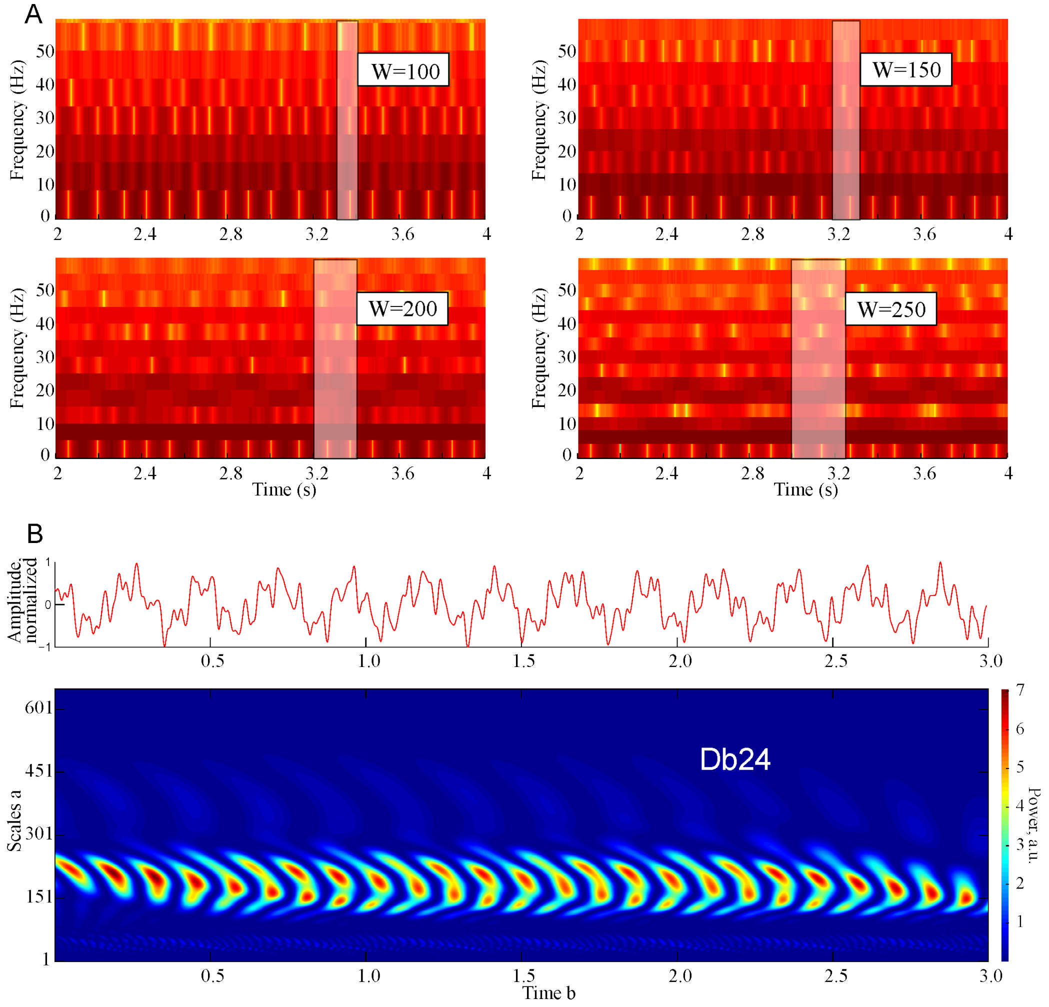

Figure 5: **Alternative methods**. **A**. The Fourier spectrograms of the same signal as shown on SFig. 4, computed for the same window widths (T_W = 0.10 sec, T_W = 0.15 sec, T_W = 0.2 sec and T_W = 0.25) do not resolve the simulated spectral waves. **B**. Wavelet spectrograms of the same signal, computed using Daubechies' wavelet of level 24, also captures the undulatory pattern of the signal but does not resolve the spectral waves.

References

[1] Grünbaum, F., The Heisenberg inequality for the discrete Fourier transform. *Applied and Computational Harmonic Analysis*, **15**, 163-67 (2003).

[2] Baker G. & Graves-Morris, P., Padé Approximants. *Cambridge Univ. Press*, (1996).

[3] Bessis, D. & Perotti, L., Universal analytic properties of noise: introducing the J-matrix formalism. *J. of Physics A* **42**(36) 365202-17 (2009).

[4] Bessis, D., Padé approximations in noise filtering. *J. Comput. Appl. Math.*, **66**, 85-88 (1996).

[5] Perotti, L., Vrinceanu, D., & Bessis, D., Enhanced Frequency Resolution in Data Analysis. *Amer. J. Comput. Math* **3**, 242-251 (2013).

[6] Perotti, L., Regimbau, T., Vrinceanu, D. & Bessis, D., Identification of gravitational-wave bursts in

high noise using Padé filtering. *Phys. Rev. D*, **90**, 124047-55 (2014).

[7] Perotti, L. & M. Wojtylak, M., (2018), Matrix methods for Padé approximation: Numerical calculation of poles, zeros and residues. *Linear Algebra and its Applications*, **548**, 95122

[8] Steinhaus, H., Über die Wahrscheinlichkeit dafuer dass der Konvergenzkreis einer Potenzreihe ihre natuerliche Grenze ist. *Mathematische Zeitschrift* **31**, 408-416 (1929).

[9] Froissart, M., Approximation de Padé: application la physique des particules élémentaires. *CNRS RCP Programme* **29**, 1-13 (1969).

[10] Gilewicz, J. & Pindor, M., Padé approximants and noise: A case of geometric series. *J. Comput. Appl. Math* **87**, 199-214 (1997).

[11] Gilewicz, J. & Kryakin, Y., Froissart doublets in Padé approximation in the case of polynomial noise. *J. Comput. Appl. Math* **153**, 235-242 (2003).

[12] Jacobsen, E. & Lyons, R., The sliding DFT. Signal Processing Magazine. *IEEE* **20**, 74-81 (2003).
